# The surgical anatomy of the axillary approach for nerve transfer procedures targeting the axillary nerve

**DOI:** 10.1007/s00276-023-03168-x

**Published:** 2023-05-22

**Authors:** Levo Beytell, Erich Mennen, Albert-Neels van Schoor, Natalie Keough

**Affiliations:** 1 Department of Anatomy, School of Medicine, Faculty of Health Sciences, University of Pretoria, Pretoria, South Africa; 2 Orthopaedic Surgeon, Mediclinic Kloof Hospital, Pretoria, South Africa; 3 Clinical Anatomy and Imaging, Department of Health Sciences, Warwick Medical School, The University of Warwick, Coventry, CV4 7AL UK

**Keywords:** Axillary nerve, Anterior approach, Nerve graft, Nerve transfer, Quadrangular space, Brachial plexus

## Abstract

**Purpose:**

The exact relational anatomy for the anterior axillary approach, targeting the axillary nerve for nerve transfers/grafts, has not been fully investigated. Therefore, this study aimed to dissect and document the gross anatomy surrounding this approach, specifically regarding the axillary nerve and its branches.

**Methods:**

Fifty-one formalin-fixed cadavers (98 axilla) were bilaterally dissected simulating the axillary approach. Measurements were taken to quantify distances between identifiable anatomical landmarks and relevant neurovascular structures encountered during this approach. The musculo-arterial triangle, described by Bertelli et al., to aid in identification on localization of the axillary nerve, was also assessed.

**Results:**

From the origin of the axillary nerve till (1) latissimus dorsi was 62.3 ± 10.7 mm and till (2) its division into anterior and posterior branches was 38.8 ± 9.6 mm. The origin of the teres minor branch along the posterior division of the axillary nerve was recorded as 6.4 ± 2.9 mm in females and 7.4 ± 2.8 mm in males. The musculo-arterial triangle reliably identified the axillary nerve in only 60.2% of the sample.

**Conclusion:**

The results clearly demonstrate that the axillary nerve and its divisions can be easily identified with this approach. The proximal axillary nerve, however, was situated deep and therefore challenging to expose. The musculo-arterial triangle was relatively successful in localising the axillary nerve, however, more consistent landmarks such as the latissimus dorsi, subscapularis, and quadrangular space have been suggested. The axillary approach may serve as a reliable and safe method to reach the axillary nerve and its divisions, allowing for adequate exposure when considering a nerve transfer or graft.

## Introduction

The axillary nerve is one of the most injured nerves in the shoulder as a result of either iatrogenic injury, shoulder dislocations, or direct trauma. Injuries to the axillary nerve greatly diminishes somatic abduction of the shoulder [13, 27]. Having multiple options for surgical access to the different parts of the axillary nerve allows for better treatment, repair, and possibly better postoperative outcomes. For isolated axillary nerve injuries, and repair thereof through a nerve transfer from one or more branches of the radial nerve, a combination between an anterior (deltopectoral) and posterior approach is typically used [4, 6]. A posterior approach has been favoured due to the direct access to target branches of both the axillary and radial nerves however, as stated by Bauer and colleagues, combining other nerve transfers during the same procedure leads to change in patient position, extended theatre time, and likelihood of additional incision sites [4]. The anterior or deltopectoral approach, although useful in accessing branches of the posterior and medial cords of the brachial plexus, has its limitations in that access to the second segment of the axillary nerve, which is considered the ideal location for nerve transfer [4].

Nerve transfer typically involves transecting a functioning nerve, or one of its branches, and either redirecting or transferring it from its original course onto an injured nerve [9], and this surgical procedure is becoming an increasingly popular method for treating nerve injuries [4]. The goal of these transfers is to either use a nerve with a lesser functional role or a branch of a nerve that already has several branches to the same structure and transferring it onto a more critical nerve that has been injured with the aim of restoring that structure’s function. Additionally, these procedures aim to reduce incidences of postoperative complications, such as local or systemic morbidity [19]. To perform a nerve transfer successfully and safely, adequate visualisation of the targeted nerves and the surrounding anatomical structures is essential [22], as well as a competently vascularised site [1] with minimal tension on the suture/s [7, 9].

As mentioned previously, several approaches to the nerve branches used for nerve transfer treatments are commonly used, however, with each approach comes a set of disadvantages. One approach, the axillary approach, described by Bertelli et al. outlines safe and effective access to the target nerves used for axillary nerve reinnervation [6]. The axillary approach requires that the patient be placed in the supine position with the arm abducted to around 90° and externally rotated. A V-shaped incision is then made over the axilla’s base just beneath the pectoralis major and extended to the area over the brachial vessels in the arm, roughly where the deltoid inserts onto the humerus. Once the incision is made, the axillary vein is retracted in a cephalad direction as needed. The area from the medial border of the latissimus dorsi to the subscapularis is then bluntly dissected to expose the subscapular artery. For further aid in locating the axillary nerve, Bertelli et al. described a musculo-arterial triangle in which the axillary nerve could be reliably found [6]. This triangle is bordered medially by the subscapular artery, laterally by the latissimus dorsi, and superiorly by the posterior circumflex humeral artery. The advantages of the axillary approach include, good visualisation of the distal axillary nerve and its branches, visualisation of the major blood vessels in the axilla, no need for sectioning of muscles [18], and it is a safe and reliable technique [6, 18]. A major disadvantage of the axillary approach, however, is the limited access to the proximal part of the axillary nerve, which is located behind the pectoralis minor [6, 11].

Although the location of the axillary nerve and its variation has been investigated in several studies [2, 6, 8, 12, 14], reports state that the segment of the axillary nerve that is anterior to the quadrangular space, is not visible through either the anterior (deltopectoral) or posterior approaches even when used in conjunction [14]. The rationale for implementing the axillary approach is that it allows for better visualisation of the structures in the axilla during the surgery. However, the precise location of the entire axillary nerve with its branches has not yet been fully defined using the axillary approach in a surgical setting [21, 25]. Therefore, this study set out to address the anatomical short comings of the axillary approach by providing a detailed anatomical description of the axillary nerve as it can be visualised through the surgical axillary approach, using visible and easily identifiable anatomical landmarks.

## Materials and methods

This study included the dissection of 102 shoulders (left and right) from 51 formalin-fixed, adult cadavers and one fresh/frozen shoulder specimen (Ethical clearance: 428/2017). All work conformed to the parameters set under the National Health Act (Act 61 of 2003). The sample consisted of 26 female and 25 male cadavers selected from anatomical dissection halls in the Department of Anatomy at the University of Pretoria. Any cadavers that displayed visible anatomical abnormalities, pathologies, or notable surgical intervention of the axillary region were excluded from the study. Age, ancestry, and sex were not considered exclusion factors.

The dissections were performed with the shoulders in an abducted and externally rotated position, although not the common position for shoulder surgery (lateral decubitus or beach chair positions), this position has been adapted and simulates the surgical position for an axillary approach in a clinical setting. Although this position was challenging to simulate in a few cases due to the rigidity caused by the embalming process, in cases where visibility or access was severely limited, only non-essential muscles in close relation to the target neurovascular structures were transected or reflected. A recent study by Matter-Parrat et al. [16] investigated whether there is a change in the position of the axillary nerve in relation to the glenoid rim when a patient is positioned in either a lateral decubitus or a beach chair position and the authors found that altering the position of the arm did not influence the position of the nerve in relation to the glenoid rim, so it is safe to assume that the nerve remains relatively consistent along its course when arm position is altered within a surgical position.

After the brachial plexus, and surrounding neurovascular structures were exposed, pins were placed into the structures and landmarks prior to any measurement being taken and ensuring the structures were kept in-situ and undisturbed. A mechanical dial calliper with an accuracy of 0.05 mm was used to take the measurements and the results were recorded into raw data sheets in Microsoft Excel (Version 1910, build 12,130.20272), which were then imported into IBM SPSS statistics (build 1.0.0.1298) and analysed for further interpretation. When placing the pins, the origin of a nerve was considered at the angle formed between the branch and the continuing trunk.

**Fig. 1:**
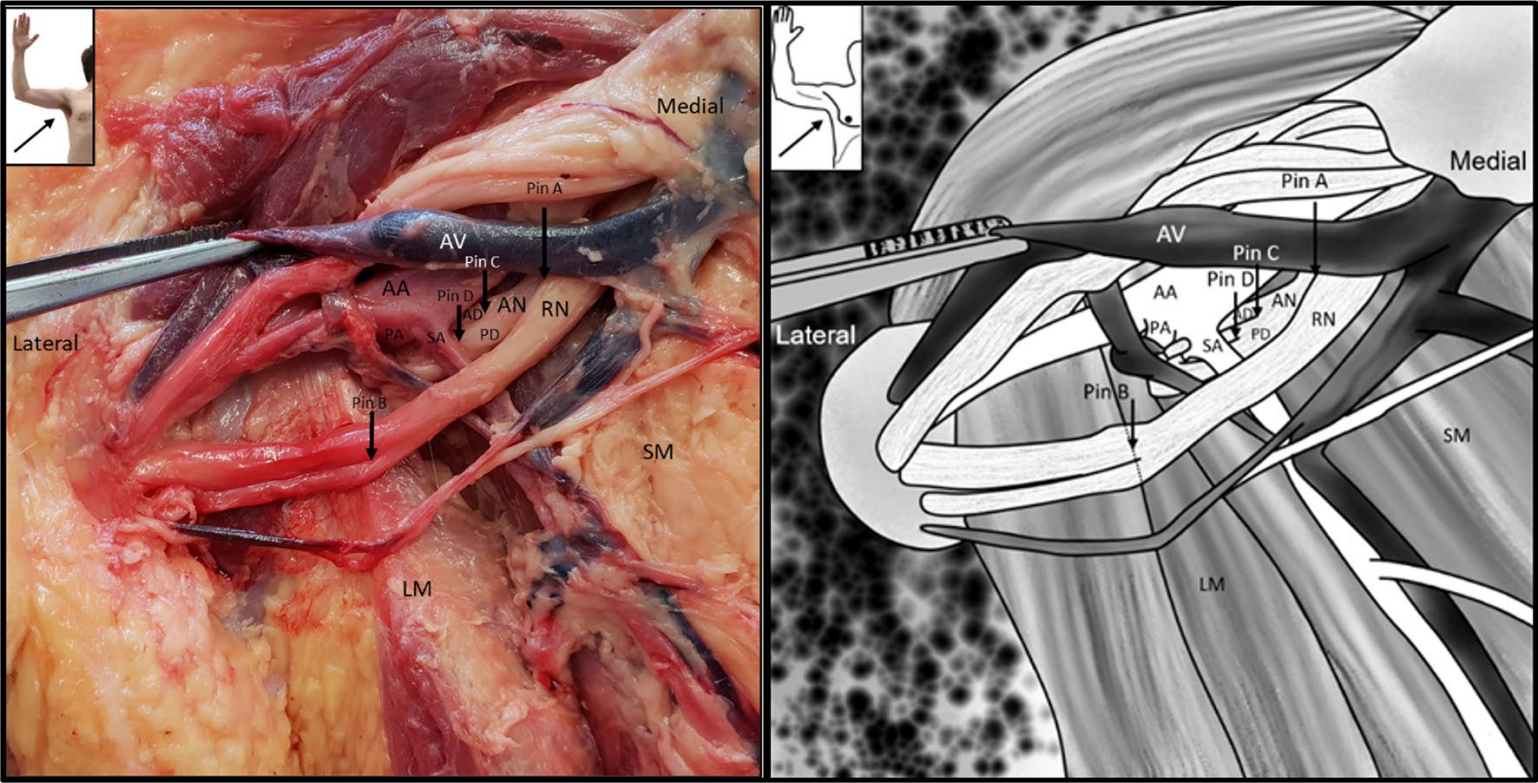
(**a**) A fresh cadaver dissection and (**b**) a diagram of a dissected axilla showing the placement of the pins used to measure the various distances. (*AN* axillary nerve, *RN* radial nerve; *AD* anterior division of axillary Nerve, *PD* posterior division of axillary nerve, *AA* axillary artery, *AV* axillary vein, *SA* subscapular artery, *PA* posterior circumflex humeral artery, *LM* latissimus dorsi muscle, *SM* subscapularis)

The following measurements were recorded (Fig. 1):

Distance A–B: the distance from the origin of the axillary nerve at the posterior cord (point A) to the anteromedial border of latissimus dorsi muscle where the radial nerve crosses over it (point B).

Distance A–C: the distance from the origin of the axillary nerve at the posterior cord (point A) to the point where the axillary nerve divides into its anterior and posterior divisions (point C).

Distance C–D: the distance from the division of the axillary nerve into its anterior and posterior divisions (point C) to the origin of the branch to the teres minor muscle along the posterior division of the axillary nerve (point D).

The diameters of the axillary nerve, as well as the diameter of its anterior and posterior divisions were also measured at their origin points. Once the measurements were taken, the pins were removed, and the axillary nerve was followed distally from its originating point at the posterior cord to the posterior side of the quadrangular space. It was then observed whether the axillary nerve divided into its anterior and posterior divisions under three conditions, (1) before entering the quadrangular space, (2) within the quadrangular space, or (3) only once it has passed through the quadrangular space. Any variations of the branches of the axillary nerve were also noted during its course.

As this study was designed to mimic the axillary approach, the borders of the quadrangular space were observed from an anterior view. The space between the inferior border of the subscapularis muscle, which is anterior to the teres minor muscle (superior border of the quadrangular space), the surgical neck of the humerus (lateral border of the quadrangular space), the latissimus dorsi muscle which is anterior to the teres major muscle (inferior border of the quadrangular space), and the long head of the triceps brachii muscle (medial border of the quadrangular space) were used for observations.

The last set of observations made were to evaluate the anatomical accuracy and validity of using the musculo-arterial triangle as described by Bertelli et al. to locate the axillary nerve and its divisions using an axillary approach [6]. The following factors were considered; (1) if the triangle was indeed formed and found without any anatomical variations obfuscating the triangle, (2) if the axillary nerve, and its divisions were seen within this triangle, (3) if only the divisions of the axillary nerve were present, and lastly (4) no presence of the axillary nerve or its branches in the triangle. The relation of the axillary and radial nerves to the subscapular artery were also noted during this process.

## Results

No significant differences were found for majority of the measurements pertaining to side or sex (*p* < 0.05) and therefore data were pooled for overall statistical analyses. All descriptive statistics are presented in Table 1. The origin of the axillary nerve, from the posterior cord of the brachial plexus to the anteromedial border of latissimus dorsi, was found to be 62.3 ± 10.7 mm and its length from origin to the point of division into its anterior and posterior divisions was 38.8 ± 9.6 mm. The only measurement that showed significant variation between males and females was the origin of the branch to teres minor, the results are presented as sex specific. The nerve to the teres minor originated around 6.4 ± 3.0 mm and 7.4 ± 2.8 mm along the posterior division of the axillary nerve measured from the division point of the axillary nerve in females and males, respectively.

**Table 1:** Descriptive statistics of distance measurements in mm (*N*  number of individuals, *Stat*. statistic, *Std. error* standard error, *SD* standard deviation, *Min* minimum, Max maximum, 95% *CI* 95% confidence interval)

Measurement	*N*	MEAN		SD	Min	Max	Range	95% CI	
		Stat	Std. error					Lower	Upper
Total A-B	98	62.33	1.08	10.70	31.72	81.90	50.18	60.21	64.44
Total A-C	98	38.76	0.97	9.57	18.64	70.29	51.65	36.86	40.65
Female C-D	47	6.39	0.39	2.68	1.94	13.10	11.16	5.63	7.16
Male C-D	50	7.41	0.39	2.78	2.90	15.52	12.62	6.64	8.18

A-B → distance from the origin of axillary nerve to anteromedial border of latissimus dorsi; A-C → distance from the origin of axillary nerve to its anterior and posterior divisions; C-D → distance from the origin of axillary nerve to origin of the branch to teres minor from posterior division of axillary nerve

The measurements of the diameters of the axillary nerve, and its anterior and posterior divisions showed great variability amongst sides and sexes at their origins. However, to allow for comparison of these diameters with previous studies, the results were combined. In this study—irrespective of side or sex—the axillary nerve had an average diameter of 4.1 ± 0.9 mm, (*n* = 98) whilst its anterior and posterior divisions had average diameters of 3.0 ± 0.9 mm (*n* = 98) and 2.8 ± 0.8 mm (*n* = 98), respectively. No correlations of note could be found between the dependent and independent variables.

A chi-square test revealed no significant difference for all observations between sides and sex except for the branching pattern of the subscapular nerve between females and males. Following the axillary nerve distally from its origin it was observed to divide into its anterior and posterior divisions within the quadrangular space in 52% of the sample (*n* = 51/98) while in the remaining 48% (*n* = 57/98), it divided before entering the quadrangular space. No occurrence of the divisions branching beyond the quadrangular space posteriorly were noted. During the axillary nerve’s course an articular branch to the shoulder joint branched off in 65 of the 98 axillae (66.3%), and in a single case, two articular branches were found to originate from the axillary nerve. In the remaining 32 (32.7%) axillae, no articular branches to the shoulder were observed with the axillary approach. Additional branches that were noted to originate from the axillary nerve directly included the lower and upper subscapular nerves. The lower subscapular nerve emerged from the axillary nerve in 39.6% of the female axillae (*n* = 19/48) and in 38% of the males (*n* = 19/50). Both the lower and upper subscapular nerves originated from the axillary nerve in 16.7% of the females (*n* = 8/48) and in 40% of the males (*n* = 20/50), considerably higher than their female counterparts.

When using the musculo-arterial triangle described by Bertelli et al. [6] to find the axillary nerve it was observed that the axillary nerve and/or both its anterior and posterior divisions were found within the described parameters of the triangle in 60.2% of the sample (*n* = 59/98). In 7 cases (*n* = 7/98; 7.1%) either the anterior or posterior division of the axillary nerve was found within the triangle and in 18 cases (*n* = 18/98; 18.4%) neither the axillary nerve nor its divisions were located within the triangle. Interestingly, in 14 of the 98 axillae (14.3%) the variation of the origin of the subscapular artery (high up on axillary artery) and/or the origin of the posterior circumflex humeral artery (from the subscapular artery), prevented the full appreciation and identification of the musculo-arterial triangle.

Regarding the relationship of the neurovascular structures in this region with this axillary approach, the axillary nerve was generally found posterior to the subscapular artery as seen in 80.6% of the sample (*n* = 79/98) axillae or superior to the artery as noted in 18.4% (*n* = 18/98) of the sample. There was a single case where the subscapular artery was found to originate more distal from the axillary artery, causing the axillary nerve to be found medial to it. The radial nerve was found anterior to the subscapular artery in 65.3% of the sample (*n* = 64/98) and posterior to it in 30.6% (*n* = 30/98) of the sample. It was rarely found superior to the subscapular artery and this variation was only found in 4 of the axillae (*n* = 4/98; 4.1%).

## Discussion

The major findings of this study demonstrate that the axillary nerve and its branches can reliably be located using surrounding soft tissue landmarks, such as the anteromedial border of latissimus dorsi, the subscapularis muscle, and the quadrangular space, when using the axillary approach for nerve transfers/grafts.

### Axillary nerve course: overview

The axillary nerve is one of the branches arising from the posterior cord of the brachial plexus, with a common root value of C5 and C6. It is a somatic nerve known to innervation both the teres minor muscle and the deltoid muscle as well as provide cutaneous supply to an area of skin over the lateral shoulder. According to Duparc et al. [8] the axillary nerve can be anatomically divided into 5 segments with the first segment representing its origin from the posterior cord of the brachial plexus till it reaches the inferior border of the subscapularis muscle and spans a length of approximately 30.5 mm; this would correlate with the point just before entering or traversing the quadrangular space. Along this section, the axillary nerve can be described as having an inferior and oblique course in relation to the subscapularis muscle [2]. At this point as well, the axillary nerve is bound within 2 possible triangles whose borders are defined by (1) pectoralis minor, coracobrachialis, and axillary artery [23] and (2) the medial edge of coracobrachialis, the lateral edge of pectoralis minor and an imaginary horizontal line connecting the superior edge of the latissimus dorsi tendon to the coracobrachialis [2]. The second part then extends around the inferior border of subscapularis, coursing in a posterior direction, till it reaches the anterolateral border of the tendon of the long head of triceps muscle spanning an approximate length of around 14.2 mm; correlating almost with it position within the quadrangular space [8]. The third part further extends from the long tendon of triceps to the surgical neck of the humerus and spans approximately 11.5 mm [8]. The fourth part continues around the surgical neck until reaching the deltoid muscle (around 28.5 mm) where it continues as the fifth part within the actual deltoid muscle [8]. This segmental breakdown provides an excellent overview of the anatomy of the nerve along its complex course from origin to termination.

### Axillary nerve and branches: lengths

To easily locate the parts or segments of the axillary nerve within an anterior approach surgical setting and within a limited surgical access space, this paper aimed to provide surgical landmarks for this purpose and relate these to the already described format by Bertelli et al. [6]. From this surgical approach (anterior approach) and in terms of the locating the actual origin point of the axillary nerve, from the posterior cord, this study found that it can be located around 62 mm (SD: 10.7 mm; CI 60.2–64.4 mm) from the anteromedial border of the latissimus dorsi muscle. The narrow confidence interval indicates that this distance is consistent, which will aid in reliably predicting the location of the axillary nerve’s origin. These results vary greatly compared to what Bertelli et al. [6] found in their study, who found the same measurement to be approximately 49.6 mm with a wide range of between 28.2 mm and 70 mm; no confidence intervals provided. The sample size difference between the current study (*n* = 98) and the study of Bertelli (*n* = 20) is significant, which may account for the difference noted, however, it should also be noted that the two studies made used of different population groups, another factor that may present with the variation noted between the two sample groups.

When considering the length of the axillary nerve from its origin to its division into anterior and posterior branches (posterior being more superficial to the anterior branch [3]), the current study demonstrated similar results (distance = 38.8 ± 9.6 mm; CI − 36.9 to 40.6 mm) as those presented by Stecco et al. [20] (distance = 44.9 ± 10.8 mm). It was noted that during dissection, the proximal part of the axillary nerve was difficult to reach through the axillary approach, however knowing this average distance can be helpful for surgeons making decisions involving the tension on the suture between nerves, which plays a critical role in recovery [9], or the length of the nerve that can be used in nerve transfer or grafting procedures. Considering Bertelli et al. [6] recommended using a combined anterior (deltopectoral) and axillary approach, instead of a posterior approach, for nerve injuries repaired through nerve grafting, knowledge of the proximal part of the axillary nerve is vital when this combined approach is used.

Various studies have found different branching patterns of the axillary nerve, however, the nerve to the teres minor muscle is most consistently found as the first branch from the posterior division of the axillary nerve [5, 6, 20, 28], or according to Duparc et al. [8] from the second segment of the axillary nerve. Similarly, this study also found the nerve to the teres minor within a short distance from the origin of posterior division of the axillary nerve, except for one case where the nerve to the teres minor muscle originated more proximal, from the axillary nerve directly. The study by Stecco et al. [20] also found this variation of the origin of the nerve to the teres minor muscle in 6 of the 16 (37.5%) axillae dissected in their sample. When collectively analysing the data and comparing it with the literature, this distance (origin of branch to teres minor from the axillary nerve division) seems to present with the most inconsistency. Additionally, the current study found significant differences between the sexes, which has not previously been reported, and established this distance in females to be 6.4 ± 2.9 mm and 7.4 ± 2.8 mm in males. When comparing this with the study by Stecco et al. [20] and Bertelli et al. [6], these authors demonstrated mean values of between 17.5 ± 7.8 mm and 19.1 ± 4.3 mm, and 12.7 mm respectively. The current study clearly showed that the teres minor branch arises from the point of the axillary nerve division much sooner than what was previously reported. These differences could possibly be explained by methodology differences. In the current study all nerves were left in-situ to the furthest possible extent and a direct measurement was taken from the origin of the teres minor branch to the division of the axillary nerve, not accounting for the turn or angle the nerve undergoes during its course, but rather the direct distance between the origin and division. Stecco et al. [20] on the other hand, first detached the deltoid muscle and along with it the axillary nerve, allowing them to straighten the nerve before taking the measurement. Although this would increase the accuracy of the “real” measurement, the goal of the current study was to maintain the integrity of the axillary approach as much as possible by not disrupting the *in-situ* position of the structures. Other possible explanations for the differences in the measurements between this study and both the study done by Stecco et al. [20] and Bertelli et al. [6] could be the larger sample size in this study, as well as the use of embalmed, versus unembalmed cadavers. However, regardless of the variation being observed, this current study confirms that if the axillary nerve can be located, together with its branching into anterior and posterior divisions, the nerve to the teres minor muscle can be readily identified and easily accessed using an axillary approach, as it can be located originating from the posterior division (normal) or originating directly from the axillary nerve (variation). In both cases, the origin of the nerve to the teres minor muscle is adequately visible from the axillary approach and lies in close relation to the division of the axillary nerve itself.

### Axillary nerve and branches: diameters

Knowledge about the diameters of the target and donor nerves plays a major role when surgeons choose to perform nerve transfer or grafting procedures and using optimal size ratios between donor and recipient nerves is key to performing successful nerve transfers [9]. It is recommended that the donor nerve must restore a single function, as well as be a pure motor or sensory nerve that is the same diameter as the recipient nerve [9]. In the context of this study, this involved documenting the diameters of the axillary nerve as well as its anterior and posterior divisions, as they can be individually repaired or used to repair other nerves. This study found the diameter of the axillary nerve to be 4.1 ± 0.9 mm, which is somewhat comparable to both Duparc et al. [8] and Stecco et al. [20] who reported the diameter to be 4.1 mm (2 mm to 6 mm) and 5.7 ± 4.4 mm, respectively. The diameter of the anterior division of the axillary nerve was found to be relatively consistent throughout the literature with the following being reported; current study (3.0 ± 0.8 mm), Stecco et al. [20] (4.0 ± 3.3 mm), Bertelli et al. [6] (2.9 mm), and Witoonchart et al. [26] (2.2 mm). The diameter of the posterior division of the axillary nerve, was also relatively consistent between studies with the following reported; current study (2.7 ± 0.8 mm), Stecco et al. [20] (2.4 ± 2.3 mm), Duparc et al. [8] (2.6 mm: range 2–3.5 mm), and Bertelli et al. [6] (2.1–2.2 mm). A note to mention is that Bertelli and colleagues specified the measurement location of the posterior division of the axillary nerve as being after the nerve to the teres minor muscle was given off, which is a slightly different measurement than the one in the current study.

### Axillary nerve and branches: relationships

When using the axillary approach, the quadrangular space is a pertinent landmark used to accurately locate the axillary nerve and its branches. This study found that the axillary nerve and its anterior and posterior divisions can be accessed, without difficulty, using the axillary approach. Based on the parameters and results of this study, the axillary nerve either branches into its anterior and posterior divisions before reaching the quadrangular space along the inferior border of subscapularis in 48% of cases or the nerve divides within the quadrangular space in the remaining 52%. These two points correlated well with segment 1 and segment 2 of the anatomical course of the axillary nerve outlined by Duparc et al. [8]. Interestingly, none of the cases followed the classical textbook description of the axillary nerve passing through the quadrangular space before dividing into its relevant branches. This was also found throughout the literature with other studies [2, 3, 6, 10, 24, 26] also reporting that the axillary nerve either divided at the quadrangular space or that it had already divided into their respective muscular branches to the deltoid muscle before the quadrangular space. The fact that the axillary nerve does not pass through the quadrangular space before branching is an important aspect for surgeons to consider when approaching the axillary nerve and its branches using the axillary approach and would make identifying the nerve easier than when trying to locate it using a posterior approach.

### Axillary nerve and branches: variations

Another aspect to note is that during nerve transfer or grafting procedures using an axillary approach, the surgeon should be vigilant of the articular branch/s from the axillary nerve to avoid any injury to these structures. The current study showed that in majority of the sample (67.3%; *n* = 66/98) either one or two articular branches coursed to the joint capsule from the axillary nerve directly. However, in 32.7% (*n* = 32/98) of the sample no articular branches were seen originating directly from the axillary nerve. Like this study, other studies also reported on finding articular branches either originating from the axillary nerve [8] or from one of its divisions [6, 25, 28]. The variation in origin of these articular branches should be considered when deciding to use the posterior or anterior divisions of the axillary nerve for nerve transfer or grafting procedures.

While studying the branches of the axillary nerve in this study, the lower and/or upper subscapular nerves were found to originate distally from the axillary nerve itself, instead of from the posterior cord as per textbook descriptions. In general, 43% (*n* = 21/48) of females and 22% of males (*n* = 11/50) had no branches originating from the axillary nerve before it divided into its anterior and posterior divisions. However, in around 39.6% (*n* = 19/48) of females and 38% (*n* = 19/50) of males, a singular lower subscapular nerve was found to originate from the axillary nerve. Both the lower and upper subscapular nerves were found to originate from the axillary nerve in 8 females (16.7%) and 20 males (40%). A study by Muthoka et al. [17] that investigated the variations of the posterior cord of the brachial plexus in a Kenyan population, found similar variations in the origins of the lower and upper subscapular nerves. The authors found this variation in more than half their sample with the lower subscapular nerve originating from the axillary nerve in 57.3% (*n* = 43/75) and the upper subscapular nerve originating from the axillary nerve in 10.7% (*n* = 8/75) of the sample. In the study conducted by Duparc et al. [8], the authors also indicated several patterns of variation that included in more than half of them, a branch to the subscapularis muscle (ssc)—upper or lower not indicated. The study by Muthoka et al. [17], also took an in-depth investigation into the origin and course of the lower and upper subscapular nerves, including observing whether these nerves originate from a common trunk. This was not done in the current study and in these cases, the observation would have been classified as the lower subscapular nerve. Based on the literature, it is clearly seen that variations do occur, and that the lower and upper subscapular nerves can originate from the axillary nerve in some cases. The relevance of this, is that it may affect the diameter of the axillary nerve at its origin. It will also likely affect the movement allowed and length of the axillary nerve that can be utilised when undertaking nerve transfer or grafting procedures using an axillary approach. The variations in the upper and lower subscapular nerves could also open more possibilities to other nerve transfers within the axilla using the axillary approach, as the lower subscapular nerve innervates the teres major muscle via muscular branches; for more on variations, Apaydin et al. [3] provide an excellent review of the anatomy and variations of the axillary nerve.

### Axillary nerve: musculo-arterial triangle

Bertelli et al. [6] described a musculo-arterial triangle bordered medially by the subscapular artery, laterally by the latissimus dorsi muscle, and superiorly by the posterior circumflex humeral artery. This triangle was described to reliably aid in locating the axillary nerve, using the axillary approach. The results of the current study only found the axillary nerve present in this triangle in 60.2% (*n* = 59/98) of the sample. In the remaining sample (*n* = 39/98; 39.8%), 7 only had one of the axillary nerve divisions in it, 18 had nothing in the triangle, and in the remaining 14 the musculo-arterial triangle was not formed and could not be identified. The main reason there was no identifiable triangle in 18 axillae, was due to the arterial variations concerning the origins of the subscapular and posterior circumflex humeral arteries noted in the sample, which lead to the distortion or absence of the pre-defined arterial border.

Based on the variations of the blood vessels involved, it was often found that the shape of the “triangle” presented more like an inverted U. With the arm abducted the subscapular artery was often seen travelling parallel to the latissimus dorsi muscle and eventually terminating as the circumflex scapular and thoracodorsal arteries. If present within the borders of these three structures, the axillary nerve was found to lie in close relationship to the posterior circumflex humeral artery. The difference between this study and the study by Bertelli could be partially due to sample size resulting in a possible type 2 error (missed detection). The frequency of the axillary nerve being found within this triangle is important, as surgeons may encounter variations during surgery and therefore knowledge of these potential variations will better equip the surgeon if these are encountered in a clinical setting. Although there might not always be a clearly defined “triangle”, the results of this study suggest that the axillary nerve predominantly courses between the three musculo-arterial landmarks, and they can be used as a reliable indicator of the position of the axillary nerve. Similarly, the axillary nerve was found in most cases to course posterior to the subscapular artery, which could be used as an indicator of the position of the axillary nerve, however the radial nerve was also found to course posterior to the subscapular artery in almost a third of cases, once again proving that the surgeon should be aware that these variations do occur and should not be always rely on a single landmark as a sole indicator of the position of a nerve.

## Limitations

The main limitation of this study was the use of formalin-fixed cadavers, the rigidness caused from the embalming process did not allow the limbs to have full range of motion and made placing the arm in the abducted and externally rotated position difficult. This could have influenced the position of important structures within the axilla. In these cases, transecting non-essential muscles allowed for greater range of motion of the upper limbs.

## Conclusion

Using the axillary approach, the axillary nerve and its branches are readily accessible especially where the axillary nerve divides into its anterior and posterior divisions. It is difficult to reach the point of origin of the axillary nerve at the posterior cord or the terminal muscular branches to the deltoid muscle. Using the musculo-arterial triangle proposed by Bertelli et al. [6] is a relatively successful method in locating the axillary nerve, however, variations in the origin of the arterial components of the triangle should be considered. Other variations as seen in the origin of the lower and upper subscapular nerves originating from the axillary nerve, could lead to possible other repair options through nerve transfer or grafting procedures using the axillary approach. Landmarks such as the latissimus dorsi muscle, subscapular muscle and quadrangular space was found to be in close relation to the axillary nerve and could therefore make for an additional or alternate landmark when identifying the nerve. These defined points can potentially aid in locating the axillary nerve and its branches in other shoulder surgeries such as RSA procedures in which the elongation of the neurovascular structures is of importance and needs to be monitored to prevent iatrogenic injuries [15]. Regardless of the end goal of the procedure that requires access to the content of the axilla, this study showed that the axillary approach can provide adequate exposure of the axillary nerve, or its branches, for nerve transfer or nerve grafting procedures.

## Data Availability

The descriptive quantitative and qualitative data results to support the findings of this study are included within the article. Full datasets used and analyzed during the current study are available from the corresponding author on reasonable request.
